# Infiltrative Hepatocellular Carcinoma: Transcatheter Arterial Chemoembolization Versus Hepatic Arterial Infusion Chemotherapy

**DOI:** 10.3389/fonc.2021.747496

**Published:** 2021-12-16

**Authors:** Chao An, Mengxuan Zuo, Wang Li, Qifeng Chen, Peihong Wu

**Affiliations:** Department of Minimal Invasive Intervention, Sun Yat-sen University Cancer Center, State Key Laboratory of Oncology in South China, Collaborative Innovation Center for Cancer Medicine, Guangzhou, China

**Keywords:** infiltrative hepatocellular carcinoma, hepatic arterial infusion, transarterial chemoembolization, ALBI, albumin-bilirubin, OS, overall survival

## Abstract

**Aims:**

To compare the effectiveness, safety, and survival outcomes in patients with infiltrative hepatocellular carcinoma (HCC) who underwent hepatic arterial infusion chemotherapy (HAIC) and transarterial chemoembolization (TACE).

**Methods:**

A total of 160 patients with infiltrative HCCs who underwent initial TACE (n = 68) and HAIC (n = 92) treatment from January 2016 to March 2020. We applied the propensity score matching (PSM) to adjust for potential imbalances. The overall survival (OS), progression-free survival (PFS), objective response rate (ORR) and disease control rate (DCR) were compared between two groups. Multivariate analysis was evaluated through the forward stepwise Cox regression model and β coefficients was applied for the nomogram construction.

**Results:**

The median follow-up duration for the study population was 20.8 months. After PSM, the median OS and PFS in the HAIC group were significantly higher than those in the TACE group (OS, 13.3 vs 10.8 months; *p* = 0.043; PFS, 7.8 vs 4.0 months; *p* = 0.035) and the ORR and DCR in the HAIC group were significantly higher than those in the TACE group (ORR, 34.8% vs 11.8%; *p* = 0.001; DCR, 54.3% vs 36.8%; *p* = 0.028). A nomogram model comprising albumin-bilirubin grade, treatment responses, sessions, and treatment modalities, showed good predictive accuracy and discrimination (training set, concordance index [C-index] of 0.789; validation set, C-index of 0.757), which outperformed other staging systems and conventional indices.

**Conclusion:**

HAIC improve significantly survival compared to TACE in patients with infiltrative HCC. A prospective randomized trial is ongoing to confirm this finding.

## Highlights

- HAIC is a safe and effective treatment in infiltrative hepatocellular carcinoma that can significantly improve survival outcomes compared with TACE.
- ALBI grade, treatment sessions, objective responds, and treatment modality (HAIC and TACE) significantly affected overall survival of patients with infiltrative hepatocellular carcinoma.
- We developed and validated a nomogram model to identify and stratify the patients with infiltrative hepatocellular carcinoma that could benefit more from two types of intra-arterial therapy.

## Introduction

Hepatocellular carcinoma (HCC) is the fourth most common malignancy resulting from hepatitis viral infections [hepatitis B virus (HBV) or hepatitis C virus (HCV)] and the third leading cause of cancer-related deaths globally (1–3). HCC is a heterogeneous disease mainly due to the effects on hepatic function and the tumor burden. For some time now, tumor burden plays an important role in tumor staging that helps physicians assess prognosis and make treatment decisions. On the basis, the multiple international guidelines for HCC [i.e., the American Joint Committee on Cancer (AJCC) and the Barcelona Clinic Liver Cancer (BCLC) staging systems] were established successively (4, 5). However, the HCC classification depends not only on tumor appearance but also on the aggressiveness of the tumor behavior. The infiltrative HCC as a morphological subtype of the tumor has aggressive abilities that are closely related to the dismal prognosis, and accounts for approximately 7% -14% of all HCC cases (the definition of infiltrative HCC see **Supplementary Information**) **(** 6). However, this rare tumor type still stays out of tumor staging even when employing the staging systems mentioned above (7, 8).

The infiltrative HCC is characterized by microscopic lesions spreading in the liver parenchyma and blood vessels throughout the whole liver. The imaging presentation includes incomplete or missing capsule, poor demarcation, and the usual occurrence with portal vein tumor thrombus, often presenting a diagnostic challenge for detecting cross sectional imaging, especially under the cirrhosis background (8). Because of the nature of infiltrative HCC, including the large size, the diffuse nature, and the propensity for the involvement of blood vessels, the treatment options available are limited and exclude surgical resection, liver transplantation, and thermal ablation. Recently, intra-arterial therapy (IAT) [i.e., transarterial chemoembolization (TACE) and ^90^Y radioembolization] summarized in **(** **Table E1** **)** were applied in the infiltrative HCC and the median survival time varied from 5.7 to 16.2 months (9–14). Moreover, hepatic arterial infusion chemotherapy (HAIC) has been used increasingly in intermediate and advanced HCC as an effective and safe transcatheter chemotherapy (15, 16), and our team has designed and confirmed that the HAIC of FOLFOX (oxaliplatin plus fluorouracil and leucovorin) regime (HAIF) for advanced HCC, which achieved satisfactory and better survival outcomes compared with sorafenib (17). However, until now the comparison of effectiveness and safety between HAIC and TACE for infiltrative HCC remain unclear.

This study compared the survival outcomes and safety of HAIC with TACE treatment in patients with infiltrative HCC by controlling the underlying selection bias across two treatment groups. We also developed a nomogram model to identify and stratify the patients that could benefit more from two types of IAT.

## Materials and Methods

This retrospective study obtained approval from the Institutional Review Board of Sun Yat-sen University Cancer Center and was conducted following the principles of the Declaration of Helsinki. Due to retrospective nature of the study, the requirement for written informed consent was waived. The article-related data were uploaded into the Research Data Deposit database (www.researchdata.org.cn, RDD:2021001941).

### Patients Enrollment

**Figure 1:**
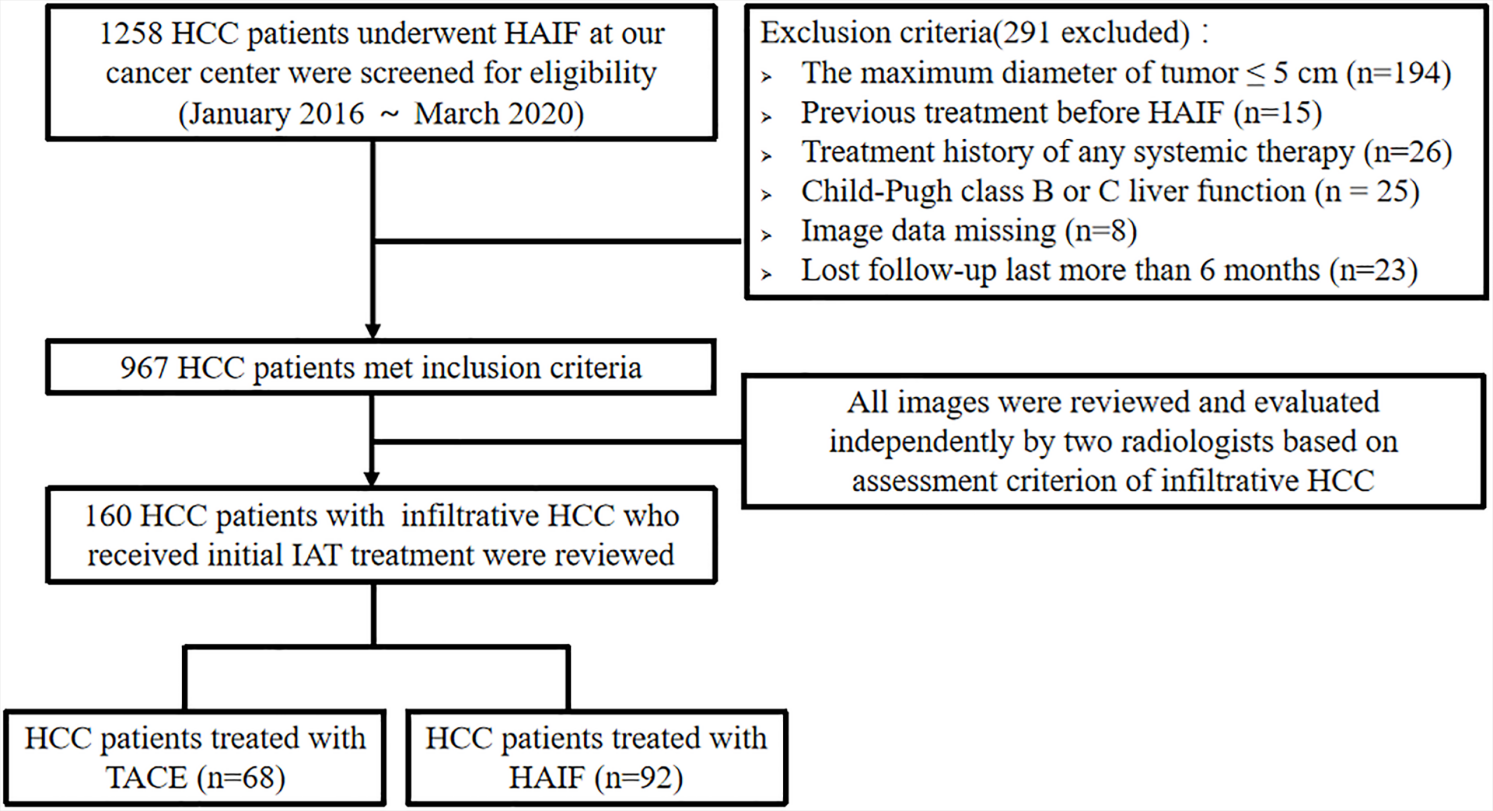
Flow diagram showing the patients with infiltrative HCC enrolled in the study.

Between January 2016 and March 2020, 1,258 consecutive patients with HCC were reviewed in our hospital’s medical database. All of the HCCs were diagnosed on the basis of the European Association for the Study of Liver (EASL) (18) and the American Association for the Study of Liver Disease (AASLD) guidelines (19). Pre-treatment images (dynamic CT, MRI) were reviewed and evaluated independently by two radiologists (L.Z.L., with 20 years of experience, and J.Z., with 8 years of experience) who were blinded to clinical procedures to confirm infiltrative HCC type according to the assessment criteria. Patients with advanced HCC were recommended for first-line multi-targeted tyrosine kinase inhibitors (TKIs) treatment, including sorafenib or lenvatinib. For the patients refusing TKI treatment due to the high financial burden, IATs were recommended as an alternative opinion according to previous studies. Moreover, several patients were confused and physicians made the final decision. Among them, 160 patients with infiltrative HCCs (16 females and 144 males; mean age, 50.9 ± 11.8 years) received IATs, including two interventional methods (TACE and HAIC) as the initial treatment. The inclusion criteria were as follows: *(a)* age 18–75 years; *(b)* Eastern Cooperative Oncology Group (ECOG) performance status < 2; *(c)* Child-Pugh class A liver function. The exclusion criteria were as follows: *(a)* patients underwent any treatment before IAT; *(b)* history of any systemic therapy; *(c)* HCC combined with other malignancies; *(d)* Child-Pugh class B or C liver function; *(e)* clinical and imaging data missing; *(f)* lost to follow-up > 6 months. **Figure 1** demonstrates the exclusion and inclusion criteria as well as patient enrollment pathways.

### HAIC and TACE Procedure

HAIC and TACE procedures have been described in a previous report (17, 20). All procedures were performed using digital subtraction angiography (Philips, type FD 20 1250 mA, Amsterdam, Netherlands). The artery sheath catheter was inserted into the femoral artery using the modified Seldinger technique. A 5-Fr Yashiro catheter (Terumo, Tokyo, Japan) was advanced into the celiac trunk and superior mesenteric artery to assess the feeding hepatic artery. A 2.7-Fr micro-catheter (Terumo, Tokyo, Japan) was inserted in the feeding artery. 1) in the HAIC group, and all chemo-drugs were given by hepatic arterial infusion through the micro-catheter. A modified FOLFOX6 regimen, including oxaliplatin (130 mg/m^2^ infusion for 3 h on day 1), leucovorin (200 mg/m^2^ for 3–5 hours on day 1), and Fluorouracil (400 mg/m2 in bolus, and then 2,400 mg/m^2^ continuous infusion 46 h) was applied. Treatment was repeated every 21 days and commonly 4–6 cycles unless intrahepatic lesions progressed or toxicity became unacceptable. 2) In the TACE group, the feeding artery was selected or super-selected whenever possible. Emulsion, which consisted of 10–20 ml lipiodol, 30–50 mg lobaplatin, and 20–40-mg epirubicin was injected slowly until the offending vessel occluded. If necessary, embolization using gel foam mixed with contrast medium was injected to reduce the residual blood flow until there was no longer any tumor staining after repeat angiography.

### Follow-Up Protocol

**Figure 2:**
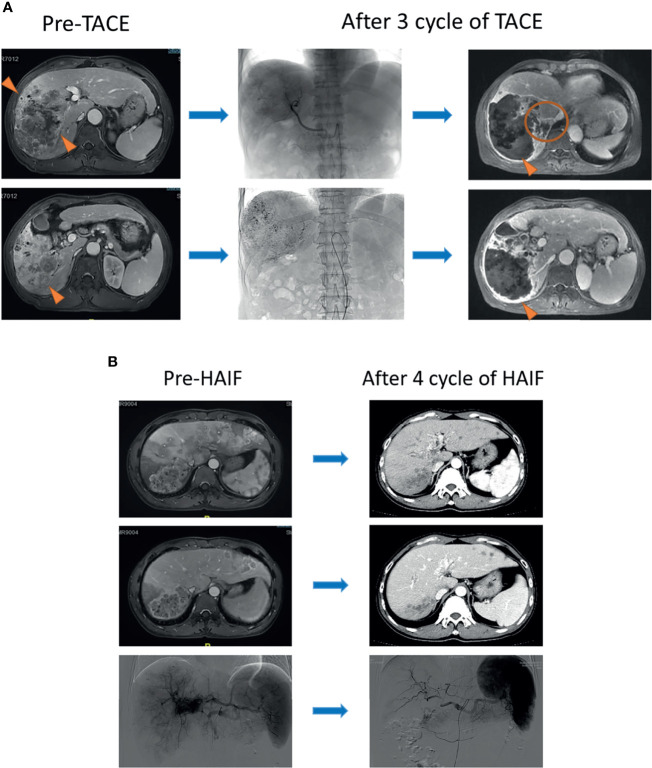
**(A)** A 57-year-old male patient was diagnosed with infiltrative HCC by contrast MRI scanning and received three-cycle TACE. Obvious necrosis appeared in the center of the tumor after therapy (triangle), but the marginal lesions became enlarged homogeneously (circle). **(B)** A 46-year-old male patient with multiple lesions was diagnosed with infiltrative HCC. Portal vein fistula was revealed at the arterial phase of the MRI scanning image. After a four-cycle HAIC, all of the lesions in the liver apparently shrank (triangle).

Routine contrast-enhanced images including computed tomography (CT) or magnetic resonance imaging (MRI), serum tumor, and hepatic function markers (α-fetoprotein, [AFP]; albumin and total bilirubin) were obtained within 1 week before and after treatment. Moreover, these examinations were assessed at 1–3 months after IAT therapy at the first year, and every 6 months follow-up after that. If suspecting metastasis was encountered, chest CT, whole-body bone scans, or positron emission tomography (PET)-CT were performed selectively. Follow-up medical records of TACE and HAIC are shown in **Figure 2**. In the whole follow-up procession, if the diameter and number of target lesions was reduced significantly after IATs, the sequential local treatment including surgery, local ablation and stereotactic body radiation therapy was considered. If the progression of target lesions occurred, the TKIs was considered. 

### Clinical Outcomes Assessment

Patients were censored at the last follow-up date (January 31, 2021). Treatment response (TR) of ITA was assessed by dynamic CT or MRI based on modified Response Evaluation Criteria in Solid Tumor (mRECIST), including complete response (CR), partial response (PR), stable disease (SD), and progression disease (PD), which was performed every 4–6 weeks. In this study, we compared three endpoints between the TACE group and the HAIC group. The primary endpoint was overall survival (OS) and progression-free survival (PFS). OS was calculated from the date of initial treatment to the date of death of any cause or deadline for follow-up. PFS was measured from the initial treatment until tumor progression, death, or deadline for follow-up. The second endpoint was the objective response rate (ORR) and disease control rate (DCR). ORR was defined as the percentage of patients with CR and PR lasting more than 4 weeks from the first radiological confirmation. DCR was defined as the percentage of patients with CR, PR, and SD. The third endpoint was adverse events (AEs) occurring during the ITA procedure (21).

### Statistical Analysis

Statistical analysis was undertaken using the RMS package of the R software version 3.6.3 (http://www.r-project.org/). The quantitative data were expressed as mean ± standard deviation or median with interquartile range (IQR), and qualitative data were expressed as frequencies. Continuous variables were analyzed using the two samples *t* test if the assumption of normality was satisfied; otherwise, the Wilcoxon rank-sum test was used. Categorical variables were analyzed using the χ^2^ test. Inter-observer agreement for treatment response was analyzed using Cohen’s kappa statistics. We applied the propensity score matching (PSM) approach based on a 1:1 ratio to the Kaplan-Meier method and Cox models for outcomes to adjust for potential imbalances in treatment assignment. Univariate and multivariate analyses of independent risk factors were evaluated through the forward stepwise Cox regression model. Cox-model-derived β coefficients were applied for nomogram construction to assess the relationship between prognosis and selected variables. All tests of significance were two-sided, and a P-value < 0.05 was interpreted to carry statistical significance.

## Results

### Study Population

A total of 160 patients with large infiltrative HCCs underwent initial TACE (n = 68), and HAIC (n = 92) treatment from January 2016 to March 2020 met the enrolled criteria. The baseline characteristics between pre-match and post-match cohorts stratified by therapeutic schedule are outlined in **Table 1**. Standardized mean differences in the pre-match cohort showed significant differences in HBV, tumor number, vascular invasion, metastasis, prothrombin time (PT), and international normalized ratio (INR). After PSM adjustment, all variables achieved adequate balance. As of January 31, 2021, the study population’s median follow-up duration was 21.2 months (IQR, 5.4, 52.7 months) in the TACE group and 20.6 months (IQR, 7.9, 50.7 months) in the HAIC group. The observed death events were 79.4% (54/68) and 83.7% (77/92) in the TACE group and HAIC group, respectively. In the TACE group and HAIC group, 76.5% (52/68) and 67.4% (62/92) progression events were observed, respectively.

**Table 1:** Baseline characteristics between TACE group and HAIC group.

Variables	Before PSM			After PSM		
	HAIC (N = 92)	TACE (N = 68)	P value	HAIC (N 68)	TACE (N = 68)	P value
**Demographics**						
Age (y), mean ± SD	50.2 ± 11.3	51.1 ± 13.1	0.673	51.6 ± 9.8	51.1 ± 13.1	0.785
Gender			0.915			1.000
Female	9 (9.8)	7 (10.3)		7 (10.3)	7 (10.3)	
Male	83 (90.2)	61 (89.7)		61 (89.7)	61 (89.7)	
PS			0.137			1.000
ECOG 0	84 (91.3)	66 (97.1)		66 (97.1)	66 (97.1)	
ECOG 1	8 (8.7)	2 (2.9)		2 (2.9)	2 (2.9)	
BMI, mean ± SD	21.4 ± 3.0	21.8 ± 3.7	0.509	22.5 ± 4.2	21.8 ± 3.7	0.114
Comorbidities			0.970			0.795
Absence	81 (88.0)	60 (88.2)		59 (86.8)	60 (88.2)	
Presence	11 (12.0)	8 (11.8)		9 (13.2)	8 (11.8)	
HBV			0.003			0.189
Absence	19 (20.7)	3 (4.4)		7 (10.3)	3 (4.4)	
Presence	73 (79.3)	65 (95.6)		59 (89.7)	65 (95.6)	
Cirrhosis			0.522			0.825
Absence	20 (21.7)	12 (17.6)		13 (19.1)	12 (17.6)	
Presence	72 (78.3)	56 (82.4)		55 (80.9)	56 (82.4)	
CTP grade			0.058			0.573
5	65 (70.7)	62 (91.2)		60 (88.2)	62 (91.2)	
6	23 (25.0)	6 (8.8)		8 (11.8)	6 (8.8)	
7	4 (4.3)	0 (0)		0 (0)	0 (0)	
ALBI score, mean ± SD	-2.59 ± 0.40	-2.65 ± 0.38	0.416	-2.62 ± 0.55	-2.65 ± 0.38	0.512
AJCC system			0.155			0.648
IIIa	2 (2.2)	1 (1.5)		1 (1.5)	1 (1.5)	
IIIb	38 (41.3)	40 (58.8)		36 (52.9)	40 (58.8)	
IIIc	48 (52.2)	26 (38.2)		31 (45.6)	26 (38.2)	
IV	4 (4.3)	1 (1.5)		0 (0)	1 (1.5)	
**Image characteristics**						
Imaging modality			0.865			0.978
CT	46 (50.0)	32 (47.0)		33 (48.5)	32 (47.0)	
MRI	21 (22.8)	18 (26.5)		18 (26.5)	18 (26.5)	
CT and MRI	25 (27.2)	18 (26.5)		17 (25.0)	18 (26.5)	
Tumor size (cm), mean ± SD	10.8 ± 3.4	11.0 ± 3.4	0.601	10.9 ± 3.6	11.0 ± 3.4	0.633
No. of tumors			0.003			1.000
Single	20 (21.7)	20 (29.4)		20 (29.4)	20 (29.4)	
Multiple	72 (78.3)	48 (70.6)		48 (70.6)	48 (70.6)	
Discrete mass			0.923			0.607
Absence	44 (47.8)	32 (47.1)		35 (51.5)	32 (47.1)	
Presence	48 (52.2)	36 (52.9)		33 (48.5)	36 (52.9)	
Vascular invasion			0.001			0.051
Absence	20 (21.7)	31 (45.6)		20 (29.4)	31 (45.6)	
Presence	72 (78.3)	37 (54.4)		48 (70.6)	37 (54.4)	
Metastasis			0.035			0.861
Absence	40 (43.5)	41 (60.3)		40 (58.8)	41 (60.3)	
Presence	52 (56.5)	27 (39.7)		28 (41.2)	27 (39.7)	
**Laboratory findings**						
AFP (ng/ml)			0.322			0.724
<400	27 (29.3)	25 (36.8)		27 (39.7)	25 (36.8)	
≥400	65 (70.7)	43 (63.2)		41 (60.3)	43 (63.2)	
Median AST (U/L)	57.2 (22.5, 90.4)	59.0 (31.5, 101.2)	0.809	54.6 (30.1,95.8)	59.0 (31.5, 101.2)	0.798
Median ALT (U/L)	92.0 (26.8,121.4)	87.9 (22.5,118.2)	0.728	83.8 (22.4,113.8)	87.9 (22.5,118.2)	0.706
Median,TBIL (μmol/L)	16.6 (7.8, 24.5)	16.5 (7.2, 22.7)	0.128	15.7 (7.2, 24.5)	16.5 (7.2, 22.7)	0.833
ALB (g/L)	39.9 ± 4.0	40.2 ± 4.3	0.676	40.1 ± 5.8	40.2 ± 4.3	0.779
INR, mean ± SD	1.11 ± 0.11	1.05 ± 0.10	<0.001	1.06 ± 0.09	1.05 ± 0.10	0.328
PT, mean ± SD	12.7 ± 1.1	12.1 ± 1.0	0.001	12.3 ± 1.2	12.1 ± 1.0	0.147
Median PLT (10^9^)	241 (78, 321)	261 (55,352)	0.257	258 (72, 315)	261 (55, 352)	0.313

Data in bracket was percent of patients. The data in two groups were compared by using the Chi square test. Non-normally distributed data is represented by median and quartile.

PSM, propensity score match; TACE, transarterial chemoembolization; HAIC, hepatic arterial infusion chemotherapy; SD, standard deviation; BMI, body mass index; PS, performance status; ECOG, Eastern Cooperative Oncology Group; HBV, viral hepatitis type B; AFP, α-fetoprotein; ALBI, albumin-bilirubin; ALB, albumin; ALT, alanine aminotransferase; AST, aspartate aminotransferase; PT, prothrombin time; INR, international normalized ratio; TBIL, total bilirubin; PLT, platelet.

### Radiological Response Rate and Hepatic Function Change

The comparison of radiological response rates between the two groups before and after PSM is shown in **Table 2**. After PSM, the ORR and DCR were 48.5% and 70.5% in the HAIC group, respectively, which remained significantly higher than those in the TACE group (*P* < 0.001, *P* = 0.001). The ALBI score was measured from the baseline to post-treatment initiation. The ALBI score change from baseline to the end of treatment was −2.55 to −2.37 in the TACE group (*P* = 0.378) and -2.62 to −2.50 in the HAIC group. The ALBI score significantly worsened in the HAIC group (+0.18 [− 0.05 to + 0.56], *P* < 0.001) and TACE group (+0.12 [− 0.05 to + 0.43], *P* < 0.001). The ALBI score showed no significant deterioration between the two groups.

**Table 2:** Treatment responds between TACE group and HAIF group.

Assessment using mRECIST	Before PSM			After PSM		
	TACE group	HAIC group	P value	TACE group	HAIC group	P value
CR	0 (0)	0 (0)	1.000	0 (0)	0 (0)	1.000
PR	8 (11.8)	40 (43.5)	<0.001	8 (11.8)	32 (34.8)	0.001
SD	17 (25.0)	20 (21.7)	0.629	17 (25.0)	18 (19.6)	0.186
PD	43 (63.2)	32 (34.8)	<0.001	43 (63.2)	18 (19.6)	<0.001
OR	8 (11.8)	40 (43.5)	<0.001	8 (11.8)	32 (34.8)	0.001
DC	25 (36.8)	60 (65.2)	<0.001	25 (36.8)	50 (54.3)	0.028

Data in bracket was percent of patients. The data in two groups were compared by using the Chi square test.

PSM, propensity score match; mRECIST, modified Response Evaluation Criteria in Solid Tumor; TACE, transarterial chemoembolization; HAIC, hepatic arterial infusion chemotherapy; CR, complete responds; PR, partial responds; SD, stable disease; PD, progression disease; OR, objective responds; DC, disease control.

### Comparison of Clinical Outcomes Before and After PSM

**Figure 3:**
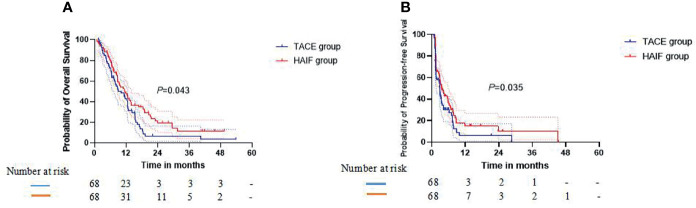
In the propensity score matching (PSM)-adjusted Kaplan-Meier analyses, **(A)** graph showing that the 1-, 2-, and 3-year cumulative OS rates in the TACE group are lower than in the HAIC group (P = 0.043). **(B)** Graph showing that the 1-, 2-, and 3-year cumulative PFS rates in the TACE group are lower than in the HAIC group (P = 0.035).

In the crude Kaplan-Meier analyses, the 1-, 2-, and 3-year cumulative OS rates were 38.2%, 8.4%, and 8.4% in the TACE group and 39.0%, 20.2%, and 8.5% in the HAIC group, respectively (**Figure 3A**), showing no significant statistical difference (*P* = 0.487). The 1-, 2-, and 3-year cumulative PFS rates were 8.5%, 8.5%, and no data (ND) in the TACE group and 19.3%, 13.6%, and 13.6% in the HAIC group, respectively (**Figure 3B**), showing a significant statistical difference (*P* = 0.033). To account for a potential bias in treatment assignments, PSM was performed. PSM-adjusted Kaplan-Meier analyses, the 1-, 2-, and 3-year cumulative OS rates were 38.2%, 8.4%, and 8.4% in the TACE group and 42.1%, 19.2%, and 13.5% in the HAIC group, respectively (**Figure 3A**), showing a significant statistical difference (*P* = 0.043). The 1-, 2-, and 3-year cumulative PFS rates were 8.5%, 8.5%, and ND in the TACE group and 19.7%, 13.4%, and 13.4% in the HAIC group, respectively (**Figure 3B**), showing a significant statistical difference (*P* = 0.035). The median OS and DFS time in the HAIC group were significantly longer than those in the TACE group (OS 13.3 vs 10.8 months; *P* = 0.043; PFS 7.8 vs 4.0 months; *P* = 0.035). Moreover, we assigned these patients with HCC into different ALBI grade subgroups. Kaplan-Meier analyses revealed comparative OS results in **Figure E1**.

### Adverse Events

No death in the two groups was directly related to IAT. The AEs between TACE and HAIC groups are shown in **Table 3**. TACE with a median of two cycles (range, 1–4) was performed during the study. The incidence of AEs in total was 49.0% in the TACE group. Among them, grades 1–2 of AEs were found in 28 of all 68 patients (41.2%), and grades 3–4 of AEs (8.8%) were leukopenia (1.5%), neutropenia (1.5%), massive ascites (2.9%), and the right shoulder back pain (2.9%). HAIC with a median of four cycles (range, 2–8) was performed during the study. AEs in the 1–2 grade was observed in 39 of all 92 patients (42.4%) in the HAIC group. Among them, the infusion-related reaction was found in seven patients (6.5%) including one (1.1%) with diarrhea, three (3.3%) with constipation, two (2.1%) with mild abdominal pain, and one (1.1%) with right shoulder back pain. AEs in 3-4 grade was found in two (2.1%) patients with leukopenia, which cause a dose reduction of HAIC.

**Table 3:** Adverse events between TACE group and HAIC group.

	TACE group (n = 68)		HAIC group (n = 92)			
	Grade 1-2	Grade 3-4	Grade 1-2		Grade 3-4	
	n (%)	n (%)	n (%)	*P* value	n (%)	*P* value
**Adverse event**	28 (41.2)	6 (8.8)	39 (42.4)	0.878	4 (4.3)	0.248
***Blood/bone marrow suppression***						
Leukopenia	8 (11.8)	1 (1.5)	3 (3.3)	0.036	NA	0.418
Neutropenia	6 (8.8)	1 (1.5)	4 (4.3)	0.248	NA	0.418
Reduced hemoglobin	2 (2.9)	NA	1 (1.1)	0.575	NA	1.000
Coagulation disorder	2 (2.9)	NA	1 (1.1)	0.575	NA	1.000
Elevated INR	7 (10.3)	NA	3 (3.3)	0.069	1 (1.1)	0.418
***Constitutional symptom***						
Weight loss	16 (23.5)	NA	14 (15.2)	0.183	NA	1.000
Fever	11 (16.2)	NA	11 (12.0)	0.444	NA	1.000
Fatigue	6 (8.8)	NA	10 (10.9)	0.670	NA	1.000
***GI disorder***						
Ascites	11 (16.2)	1 (1.5)	12 (13.0)	0.577	2 (2.2)	1.000
Diarrhea	2 (2.9)	NA	1 (1.1)	0.575	NA	1.000
Anorexia	2 (2.9)	NA	1 (1.1)	0.575	NA	1.000
Constipation	4 (5.9)	NA	NA	0.031	NA	1.000
Vomiting	6 (8.8)	NA	3 (3.3)	0.131	NA	1.000
***Pain***						
Abdominal nonspecific	3 (2.3)	NA	NA		NA	1.000
Right shoulder back	NA	2 (2.9)	1(1.1)		NA	0.179
***Laboratory abnormalities***						
Elevated ALT	26 (38.2)	2 (2.9)	31 (33.7)	0.553	1 (1.1)	0.575
Elevated AST	29 (42.6)	1 (1.5)	22 (23.9)	0.012	NA	0.418
Elevated TBIL	12 (17.6)	NA	12 (13.0)	0.420	NA	1.000
Elevated creatinine	9 (13.2)	NA	11 (12.0)	0.809	NA	1.000
Anaemia	2 (2.9)	NA	NA	0.179	NA	1.000
Others	10 (14.)	NA	5 (5.4)	0.047	NA	1.000

Data in bracket was percent of patients. The data in two groups were compared by using the Chi square test. *Data were compared by using Fisher’s exact test. TACE, transarterial chemoembolization; HAIC, hepatic arterial infusion chemotherapy; ALT, alanine aminotransferase; AST, aspartate aminotransferase; GI, gastrointestinal; INR, international normalized ratio; TBIL, total bilirubin.

### Univariate and Multivariate Analyses of OS

The results of univariate and multivariate Cox regression analyses for OS are summarized in **Table 4**. In multivariate step-wise Cox regression analysis, ALBI grade (HR: 1.652; 95% CI: 1.164, 2.345; *P* = 0.005), sessions (HR: 0.389; 95% CI: 0.248, 0.609; *P* < 0.001), TR (HR: 0.409; 95% CI: 0.255, 0.606; *P* < 0.001), and treatment modality (HR: 0.401; 95% CI: 0.259, 0.621; *P* < 0.001) significantly affected OS. If the pre-treatment variables were used in the Cox regression analysis, ALBI grade (HR: 3.652; 95% CI: 1.924, 5.316; *P* < 0.001), sessions (HR: 2.258; 95% CI: 1.680, 4.258; *P* = 0.002), and treatment modality (HR: 1.214; 95% CI: 1.107, 1.857; *P* = 0.011) significantly affected OS.

**Table 4:** Results of the univariable and multivariable Cox regression model with regard to OS.

Variable	Univariable Model		Multivariable Model			
			Before PSM		After PSM	
	HR (95% CI)	P value	HR (95% CI)	P value	HR (95% CI)	P value
Age (continuous)	0.661 (0.383, 1.133)	0.132	–	–	–	–
Gender (male)	1.459 (0.765, 2.782)	0.252	–	–	–	–
Comorbidities (presence)	1.049 (0.620, 1.775)	0.859	–	–	–	–
Tumor size (continuous)	1.225 (0.887, 1.777)	0.200	–	–	–	–
No. of tumors (multiple)	0.903 (0.577, 1.412)	0.654	–	–	–	–
HBV (absence)	1.492 (0.867, 2.567)	0.149	–	–	–	–
ALBI grade (2)	1.363 (0.965, 1.925)	0.079	1.652 (1.164, 2.345)	0.005	1478 (1.140, 2.378)	0.007
PVTT (absence)	1.402 (0.978, 2.010)	0.066	–	–	–	–
Metastasis (absence)	0.843 (0.598, 1.189)	0.331	–	–	–	–
AFP (≥400 ng/ml)	1.370 (0.945, 1.986)	0.097	–	–	–	–
Sessions (>3)	0.514 (0.354, 0.747)	<0.001	0.389 (0.248, 0.609)	<0.001	0.497 (0.268, 0.752)	<0.001
Treatment modalities	1.132 (0.769, 1.610)	0.490	0.401 (0.259, 0.621)	<0.001	0.415 (0.262, 0.771)	<0.001
HAIC						
TACE						
Treatment responds						
PR	–	–	–	–	–	–
SD	0.248 (0.157, 0.391)	<0.001	0.409 (0.255, 0.606)	<0.001	0.415 (0.252, 0.609)	<0.001
PD	0.523 (0.337, 0.812)	0.004	0.388 (0.248, 0.608)	<0.001	0.382 (0.243, 0.611)	<0.001

OS, overall survival; HR, hazard ratio; CL, confidence interval; PSM, propensity score match; TACE, transarterial chemoembolization; HAIC, hepatic arterial infusion chemotherapy; HBV, hepatitis B virus; CTP, child-turcotte-pugh; AFP, α-fetoprotein; AST, aspartate aminotransferase; ALT, alanine aminotransferase; TBIL, total bilirubin; ALB, albumin.

### Development and Validation of the Prognostic Model

**Figure 4:**
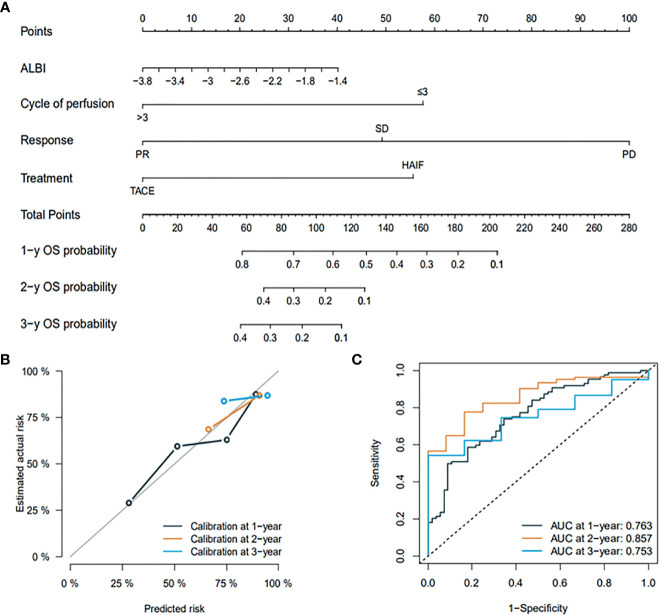
**(A)** graph showing the prognostic model for predicting 1-, 2-, and 3-year OS in all patients. **(B)** Calibration plots for the prognostic outcome. **(C)** ROC curve for internal validation.

All of the patients were assigned to the training dataset (n = 128) and the internal validation dataset (n = 32). The baseline characteristics of the training and validation dataset are shown in **Table E2**. The prognostic outcome should exceed 10 times of variable number to confirm the expected error in the predicted probabilities < 10%. In our study, 131 deaths were found, which is 32.75 times higher than four variable-related OS, including ALBI, TR, sessions, and treatment modality. A visualizable prognostic model for predicting 1-, 2-, and 3-year OS was developed and graphically presented **(** **Figure 4A** **)**. The nomogram exhibited good discrimination and continued to show good predictive accuracy and discrimination for OS in the training dataset with a C-index of 0.789 (95% CI, 0.722-0.814), and a similar result (C-index of 0.757, 95% CI, 0.717-0.787) was also found in the internal validation dataset using 1,000 bootstrap resampling analyses. The prognostic outcome’s calibration plots had good predictive value and were validated well in the training dataset internal validation dataset **(** **Figure 4B** **)**. The AUCs of 1-, 2-, and 3-year OS were 0.767, 0.857, and 0.753, respectively **(** **Figure 4C** **)**. The predictive performance and discrimination were higher than that in the predictive model comprising pre-treatment variables, conventional HCC staging, and indices **(** **Table E3** **)**.

## Discussion

In this study, 160 patients who met the enrolled criteria were selected from 1,258 patients at our cancer center in a real-world study. To the best of our knowledge, this is the first study comparing HAIC and TACE as initial therapy for large infiltrative HCCs. We found that the OS and PFS outcomes in the HAIC group were both significantly higher than those in the TACE group when the variables keep balance underwent PSM. This primary outcome suggests that HAIC treatment can offer survival benefits compared with TACE in patients with large infiltrative HCCs. The ALBI grade consists of serum albumin and bilirubin, giving an objective and evidence-based tool for assessing the hepatic function that could replace the Child-Pugh class (22–24). We found that both groups had a significant hepatic function deterioration by assessing ALBI score changes, but the deterioration degree of the TACE group was similar to that of the HAIC group. In this study, the patients with large infiltrative HCCs were subdivided into two groups based on the ALBI grade, and the survival benefits remain to be found in the HAIC group regardless of ALBI grade.

In this study, the ORR and DCR in the HAIC group were both significantly higher than those in the TACE group before and after PSM. Previous studies have revealed that high tumor burden is an independent prognostic factor for HCC. Therefore, better ORR and DCR contribute to improving survival. After PSM processing, the median OS in the HAIC group reached 13.3 months, which is superior to only 10.8 months in the TACE group (*P* = 0.035). This result is slightly lower than that of the Lyu et al. study (14.5 months) (17). The reasons may be associated with different tumor types. Given the scarce number of reports about infiltrative HCC, the available data are limited. Kichang Han et al. reported that TACE is a safe treatment option in infiltrative HCC patients with Child-Pugh class A, but its median OS time was only 5.7 months (10). However, Peter J. Kneuertz et al. found that IAT treatment including drug-eluting beads-TACE, conventional TACE, and yttrium-90 (Y-90) in infiltrative HCC patients can lead to 12 months of median OS time (9). Unfortunately, we have not found reports of sorafenib treatment in infiltrative HCC, but for advanced HCC, the median OS of patients who received sorafenib treatment was approximately 6.5 months in several phase II and phase III trials (16, 25–27).

The IAT treatment has been previously reported to offer a survival benefit to patients with infiltrative HCC, and it has been proven to exceed the therapeutic effect of the best supportive care. However, in the case of infiltrative HCC, especially large infiltrative, due to its diffuse nature and high tumor burden, it is refractory to repeat TACE. Given that infiltrative HCC is a morphologic subtype of missing complete capsule, which is commonly associated with hypervascularity or vascular abnormalities, it is difficult for the TACE procedure to block most of the supply arteries to ensure the deposition of lipiodol and drug-loaded microspheres and the direct action of chemotherapy drugs. Moreover, HAIC has been used to eradicate advanced HCC in many Asian countries. In particular, HAIC has been recommended as the first-line treatment in Japan (28). Traditional infusion chemotherapy regimens mainly depend on cisplatin combined with fluorouracil, but cisplatin has inevitable toxicities, which caused more AEs after HAIC and forced physicians to reduce the dose. Although the high-dose regimen can improve the therapeutic effect, it still cannot be used continuously. FOLFOX using oxaliplatin instead of cisplatin is a combined and classic anticancer method and proved to be effective systemically for advanced HCC. Moreover, the well-received advantage of HAIC is a lower incidence of AEs and major complications compared with systemic chemotherapy and TKIs (29, 30). The incidence of grade 1-2 (42.4%) and 3-4 AEs (2.1%) were found in our study. These results confirm further that HAIC is a safe and effective therapeutic approach for infiltrative HCC.

The prognostic nomogram model comprising three pre-treatment variables (ALBI grade, treatment modality, and sessions) and one variable of post-treatment (treatment responses) were built and validated in this study. The visual model achieved better predictive ability with C-index value of 0.789 in the training set and a C-index of 0.757 in the internal set when four variables are put into the Cox regression model simultaneously, which outperformed those in the model using variables of pre-treatment (0.722 for the training set and 0.707 for the internal set). Similarly, the results indicate a higher reliability and nomogram model’s preciseness than traditional staging systems (AJCC and BCLC staging system). Among three pre-treatment variables, selective HAIC should be recommended, and hepatic function has always been regarded as one of the key factors in predicting survival prognosis. Furthermore, > 3 cycles of IAT treatment is highly effective in terms of treatment response and survival time and should be applied as much as possible when the patient’s performance was comfortable. Moreover, TR is an independent risk factor for predicting OS, but ORR is not entirely dependent on the selection of treatment modality because HCC heterogeneity in a high degree, even in HAIC, has a higher ORR superior to TACE. Given its identification power and stability, this nomogram model can provide physicians and patients with a prognostic risk score before and after IAT treatment, thereby ensuring the patient’s follow-up and subsequent treatment.

There are some limitations to our study. First, the risk of selection bias is unavoidable in observational studies. However, this risk has been minimized by including all consecutive patients with infiltrative HCC and using PSM. Second, the cohort is a single-center, retrospective study, and the sample size is relatively small. The multi-center, large cohort, and prospective studies are necessary to design in the future to verify our results; finally, at present, there is no universally recognized evaluation criterion of infiltrative HCC, so there may be some imbalances in the inclusive population of the two treatment groups, which may cause the biased comparative results of survival outcomes.

Until now, infiltrative HCC has lacked an accurate clinical staging and well-received treatment methods. Here, we have shown that HAIC is a safe and effective treatment in infiltrative HCCs that can significantly improve survival outcomes compared with TACE. We also established a novel prognostic model that can help physicians make ITA decisions and evaluate the pre-and post-treatment variables on survival outcome. However, a multiple-center prospective clinical trial is needed to further validate this result.

## Data Availability Statement

The raw data supporting the conclusions of this article will be made available by the authors, without undue reservation.

## Ethics Statement

The studies involving human participants were reviewed and approved by The Institutional Review Board of Sun Yat-sen University Cancer Center. Written informed consent from the participants’ legal guardian/next of kin was not required to participate in this study in accordance with the national legislation and the institutional requirements. Written informed consent was not obtained from the individual(s), nor the minor(s)’ legal guardian/next of kin, for the publication of any potentially identifiable images or data included in this article.

## Author Contributions

Study concept and design: CA and MZ. Drafting of the manuscript: CA. Acquisition of data, analysis and interpretation of data: MZ. Critical revision of the manuscript: PW. Statistical analysis: QC. Study supervision: WL. All authors contributed to the article and approved the submitted version.

## Conflict of Interest

The authors declare that the research was conducted in the absence of any commercial or financial relationships that could be construed as a potential conflict of interest.

## Publisher’s Note

All claims expressed in this article are solely those of the authors and do not necessarily represent those of their affiliated organizations, or those of the publisher, the editors and the reviewers. Any product that may be evaluated in this article, or claim that may be made by its manufacturer, is not guaranteed or endorsed by the publisher.

## Abbreviations

HCC, hepatocellular carcinoma; IAT, intra-arterial therapy; TACE, transarterial chemoembolization; HAIC, hepatic arterial infusion chemotherapy; TKI, tyrosine kinase inhibitors; IPTW, inverse probability of treatment weighting; AFP, alpha-fetoprotein; AST, aspartate aminotransferase; ALT, alanine aminotransferase; ALBI, albumin-bilirubin; mRECIST, modified Response Evaluation Criteria in Solid Tumor; CR, complete response; PR, partial response; SD, stable disease; PD, progression disease; OS, overall survival; PFS, progression-free survival.
