# Supplementary material for: Infiltrative Hepatocellular Carcinoma: Transcatheter Arterial Chemoembolization Versus Hepatic Arterial Infusion Chemotherapy
Source: Front Oncol. 2021 Dec 16;11:747496. doi: 10.3389/fonc.2021.747496 (PMC8716800; doi:10.3389/fonc.2021.747496)

Infiltrative Hepatocellular Carcinoma: Transcatheter Arterial Chemoembolization versus Hepatic Arterial Infusion Chemotherapy

1. Supplementary Methods

1.1 Assessment criteria of infiltrative HCC

Assessment criteria of infiltrative hepatocellular carcinoma: 1) diagnosed with HCC; 2) incomplete or missing capsule; 3) poor demarcation and permeating appearance on cross sectional imaging; 4) hypointense on T1WI and homogeneous and mild to moderately hyperintense on T2WI of MRI scanning image; 5) initial post-contrast images may show “miliary enhancement;” and 6) usually company with portal vein tumor thrombus.

1.2 Typical imaging present of infiltrative HCC

There are multiple masses and nodules in the liver, the larger ones are located under the capsule of the right lobe of the liver. The contour is not smooth, involving the liver capsule. Furthermore, the larger cross-section area is about 85 mm × 54 mm, showing multi-nodule fusion and obviously uneven enhancement in enhanced arterial phase, decreased enhancement in the portal phase, and delayed phase (Figure 1).

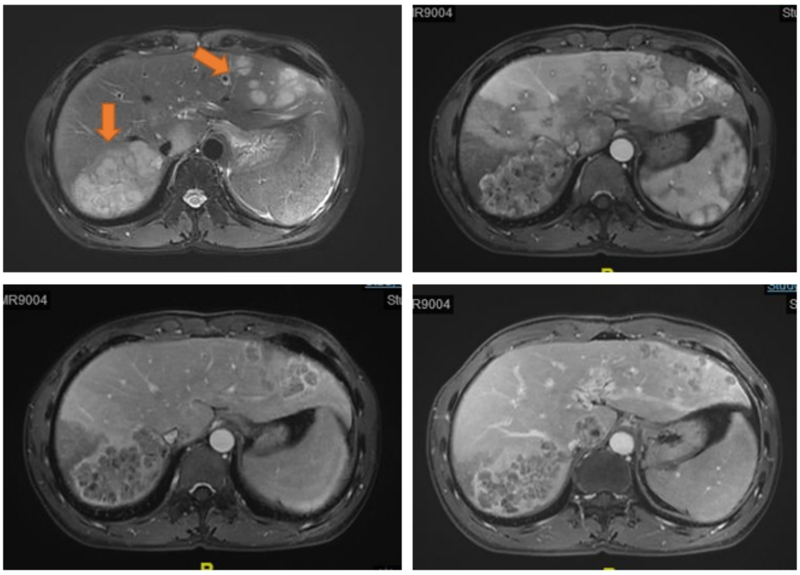

2. Supplementary Table

2.1 Table E1. The recent advances in clinical studies involving the HAIC for HCC.

2.2 Table E2. Baseline characteristics between the training set and validation set.

2.3 Table E3. Performance of models and clinical indices.

Table E1. The recent advances in clinical studies involving the HAIC for HCC.

Authors	Year	Sample size	Treatment	Size (cm)	Stage	Median OS (months)	ORR (%)
Peter J. Kneuertz [9]	2012	75	DEB-TACE /C-TACE, Y^{90} radioembolization	9.5	C	10	33.3
Kichang Han [10]	2014	46	TACE	15	C	5.7	18.0
Nima Kokabi [11]	2015	30	Y^{90} radioembolization	9.2	C	13	NA
McDevitt. et al. [12]	2017	50	Y^{90} radioembolization, Drug-eluting embolic	10.5	C	9.9/8.1	33.0
Zi-shu Zhang [13]	2019	89	DEB-TACE /C-TACE	11.2/10.7	B, C	NA	10.7/12.1
Michael J Nisiewicz[14]	2021	53	TARE	10.8	C	16.2	54.1

Table E2. Baseline characteristics between the training set and validation set.

**Variables**	**Training Set (n = 128)**	**Validation Set (n = 32)**	***P* Value**
**Demographic and history**			
Mean age ± SD (years)	50.9±10.7	49.2±10.9	0.764[a]
Sex			0.015[b]
Male	119 (93.0)	26 (78.8)	
Female	9 (7.0)	7 (21.2)	
Mean BMI ± SD (kg/m^2)	22.5±6.2	21.8±5.5	0.514[a]
Performance status			0.102[b]
0	122 (95.3)	28(87.5)	
1	6 (4.7)	4 (12.5)	
Comorbidities			0.903[b]
Absence	15 (11.7)	4 (12.5)	
Presence	113 (88.3)	28 (87.5)	
Etiology			<0.001[b]
HBV	120 (93.6)	19 (59.4)	
Other	8 (6.4)	13 (40.6)	
Cirrhosis			1.000[b]
Absence	120 (93.6)	120 (93.6)	
Presence	8 (6.4)	8 (6.4)	
CTP grade			0.800[b]
5	125 (97.7)	31 (96.9)	
6-7	3 (2.3)	1 (3.1)	
Mean ALBI score ± SD	-2.65±0.21	-2.59±0.32	0.478
Median AFP level (ng/ml)	2289.25 (3.2-121000)	3578.14(4.8-121000)	0.212[a]
**Tumor data**			
Mean maximal tumor diameter ± SD (cm)	10.8±2.3	11.1±3.4	0.788[a]
No. of tumors			0.129[b]
Single	27 (78.8)	3 (58.8)	
Multiple	101 (21.2)	29 (41.2)	
Vascular invasion			0.069[b]
Absence	50 (39.1)	7 (17.9)	
Presence	78 (60.9)	25 (72.1)	
Metastasis			0.097
Absence	69 (40.3)	12 (40.3)	
Presence	59 (59.7)	20 (59.7)	
**Laboratory findings**			
Mean albumin level ± SD (g/L)	40.3±3.3	39.0±3.5	0.492[a]
Median total bilirubin level (μmol/L)	19.3 (2.2-62.9)	18.4 (3.7-62.1)	0.971[a]
Median ALT (U/L) (range)	89.5 (8.1-182.5)	91.4 (9.6-212.3)	0.681[a]
Median AST (U/L) (range)	56.7 (12.9-199.3)	58.2 (8.8-187.6)	0.219[a]
Median platelet counts (×10^9) (range)	248 (67-367)	256 (56-385)	0.562[a]
Mean INR ± SD	1.13±0.21	1.15±0.32	0.898[a]
Sessions			0.933[b]

<3	85 (66.4)	21 (65.6)	
>3	43 (33.6)	11 (34.4)	
Complications[§]	2/128(1.6)	1/32 (3.1)	0.490[b]
Follow-up (years)			0.787[a]
Median	19.8	18.7	
Range	2.5-57.8	3.2-55.6	

Note.—Except where indicated, data are numbers of patients. Data in parentheses are percentages and were calculated using the total number of patients in each group as the denominator. SD = standard deviation. $P < 0.05$ indicates a significant difference.

[a]Student's *t* test

[b]Pearson X^2 test

§Data in parentheses are percentages.

BMI, body mass index; PS, performance status; HBV, viral hepatitis type B; AFP, α-fetoprotein; ALBI, albumin-bilirubin; ALB, albumin; ALT, alanine aminotransferase; AST, aspartate aminotransferase; PT, prothrombin time; INR, international normalized ratio; TBIL, total bilirubin; PLT, platelet.

Table E3. Performance of models and clinical indices.

**Model and clinical indices**	**C-index**	**95% CI**	***P* value***	**Wald test**	**LR test**	**AIC**
Nomogram 1 in TS	0.789	0.722, 0.814	1.000	21.24	40.21	526.47
Nomogram 1in VS	0.757	0.717, 0.787	0.515	37.51	20.78	558.56
Nomogram 2 in TS	0.722	0.685, 0.784	0.215	11.52	19.36	653.14
Nomogram 2 in VS	0.707	0.652, 0.743	0.187	10.78	15.64	689.37
TNM system	0.590	0.532, 0.628	< 0.001	11.24	10.24	798.25
ALBI grade	0.524	0.517, 0.586	< 0.001	9.78	8.64	882.47
AFP level	0.504	0.457, 0.553	< 0.001	16.63	56.47	1026.24

Nomogram 1, all variables; Nomogram 2, pre-treatment variables; TS, Training dataset; VS, Validation dataset; AIC: Akaike Information Criterion; TNM: Tumor-Node-Metastasis.

*Other models compared with Nomogram in the training set.

3. Supplementary Figure

3.1 Figure E1. Kaplan-Meier analyses show comparative OS results in different ALBI grade subgroups.

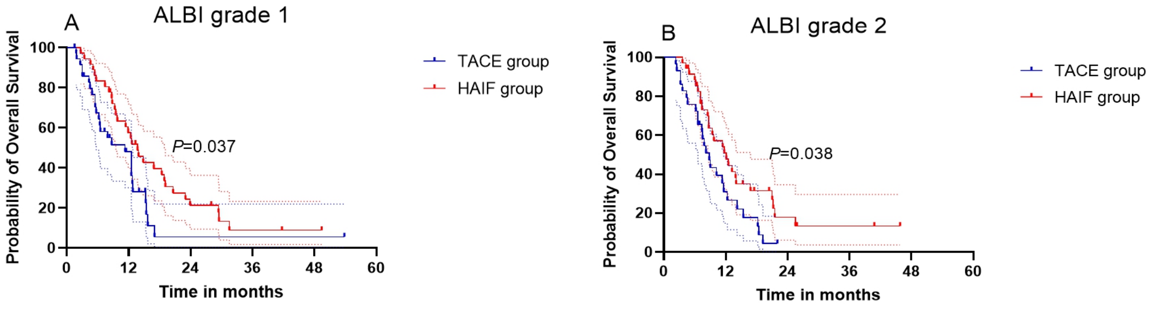

Supplement: Supplementary file 1 [file DataSheet_1.pdf]
